# Ce‐Doped Lignin‐Based Nanozyme with Heat‐Activated Hydrolase Activity

**DOI:** 10.1002/advs.202519268

**Published:** 2026-01-23

**Authors:** Xin Liu, Lijun Li, Xue Zhang, Alireza Ashori, Fubao Sun, Feng Xu, Xueming Zhang, Yao Chen

**Affiliations:** ^1^ Beijing Key Laboratory of Lignocellulosic Chemistry Beijing Forestry University Beijing China; ^2^ State Key Laboratory of Biopharmaceutical Preparation and Delivery, Institute of Process Engineering Chinese Academy of Sciences Beijing China; ^3^ China National Pulp and Paper Research Institute Co., Ltd. Beijing China; ^4^ Department of Chemical Technologies Iranian Research Organization For Science and Technology (IROST) Tehran Iran; ^5^ Key Laboratory of Industrial Biotechnology Ministry of Education School of Biotechnology Jiangnan University Wuxi China; ^6^ State Key Laboratory of Bio‐based Fiber Materials Zhejiang Sci‐Tech University Hangzhou China

**Keywords:** antibacterial, heat‐activated, hydrolase, lignin, nanozyme

## Abstract

Nanozymes are powerful alternatives to natural enzymes, yet the sustainable design of hydrolase nanozymes using renewable ligands remains underexplored. Lignin, as an abundant aromatic biopolymer, offers a promising and green ligand for constructing sustainable nanozymes. In this work, a Ce‐doped lignin‐based hydrolase nanozyme (Ce‐AL) is synthesized using Ce ions coordinated with aminated industrial lignin. The resulting Ce‐N_x_ centered Ce‐AL enable the hydrolysis of phosphate esters and even stable protein amide bonds. Notably, the hydrolase‐like activity of Ce‐AL at 100°C is 2.03 times that at 40°C, attributable to the thermally optimized Ce‐N_x_ sites within the protective lignin scaffold. The Ce‐AL effectively combats stubborn biofilms formed by Gram‐negative (*Escherichia coli*) and Gram‐positive (*Staphylococcus aureus*) bacteria by selectively hydrolyzing key proteinaceous and nucleotide components of the extracellular polymeric substances and bacterial cell walls. This work highlights lignin as a renewable platform for designing advanced, stimuli‐responsive nanozymes.

## Graphical abstract

A novel Ce‐doped lignin‐based hydrolase nanozyme (Ce‐AL) is synthesized using Ce ions coordinated with aminated industrial lignin. The Ce‐AL effectively hydrolyzes phosphate ester bonds and protein amide bonds, maintain high activity at 100°C, and successfully fights bacteria biofilms. This work achieves the valorization of industrial lignin and expands the nanozyme toolbox with a sustainable and heat‐activated hydrolase nanozyme.

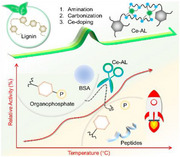

## 1 Introduction

Nanozymes, nanomaterials with intrinsic enzyme‐like activities, have emerged as powerful alternatives to natural enzymes owing to their superior stability, tunable catalytic properties, and cost‐effectiveness [1]. Based on catalytic activity types, nanozymes could be categorized into two major groups: oxidoreductases (including peroxidase, oxidase, catalase, and superoxide dismutase) and non‐oxidoreductases (such as hydrolases, urease, lipases) [2]. Among these, hydrolase nanozymes are particularly promising because they operate efficiently in aqueous environments without requiring H_2_O_2_ activation or O_2_ supply [3, 4]. Moreover, some hydrolytic nanozymes exhibit broad substrate versatility, endowing them with great potential in environmental remediation and therapeutic applications [5, 6, 7]. To date, various hydrolase nanozymes based on metal oxides, metal–organic framework (MOF), and single‐atom sites have been developed to target key biomolecules such as signaling compounds, glycosidic bonds, and extracellular DNA [8, 9, 10, 11]. Notably, certain MOF‐based hydrolase nanozymes have even demonstrated broad‐spectrum activity against complex components in bacterial biofilms [12, 13].

Nevertheless, the pursuit of sustainable design in nanozyme development, especially the exploration of renewable and eco‐friendly ligand sources, remains a significantly underexplored frontier. The valorization of abundant biomass resources (such as cellulose, lignin, and hemicellulose) offers a compelling solution to this challenge [14, 15, 16]. Leveraging these natural polymers as green building blocks for nanozymes can not only reduce reliance on non‐renewable resources but also capitalize on their inherent chemical reactivity and biocompatibility for advanced catalytic applications [17, 18, 19].

Lignin is the most abundant aromatic biopolymer on earth and is highly amenable to chemical modification [20, 21, 22]. Therefore, aminated lignin, which contains reactive alcohol hydroxyl groups and amine groups, represents a promising ligand for coordinating metal ions [15, 23, 24, 25]. In our previous studies, the Fe‐doped lignin‐based peroxidase nanozyme (Fe‐AL) [26] and CuN_x_‐centered lignin‐based laccase nanozyme (Cu‐AL) [27] were prepared using aminated lignin as the ligand. These nanozymes exhibited exceptional detection sensitivity and recyclability, enabling their successful application in H_2_O_2_ detection and phenols degradation, respectively. However, research on lignin‐based hydrolase nanozymes with promising properties has not yet been reported.

**SCHEME 1:**
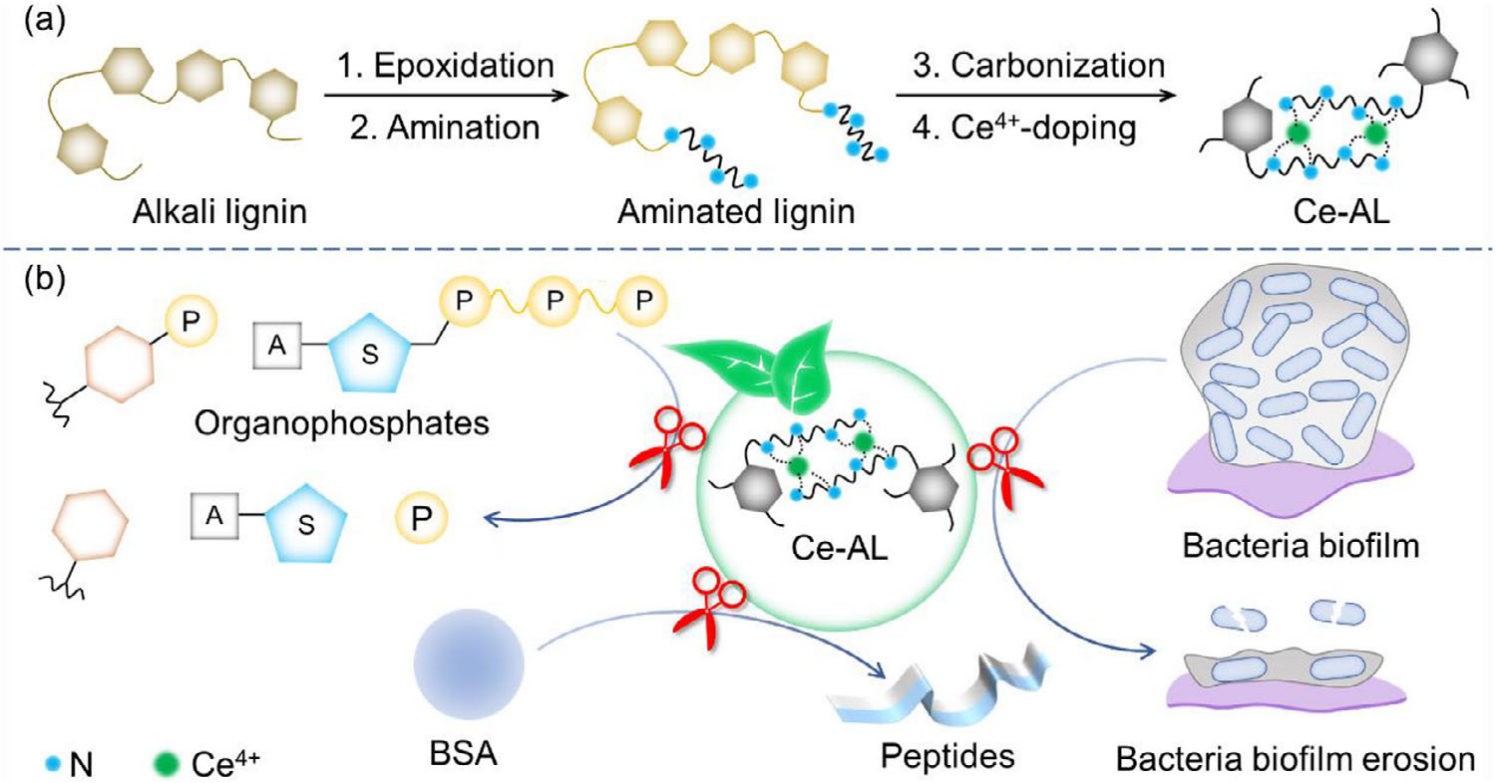
(a) Schematic illustration of the preparation of Ce‐AL; (b) Application of Ce‐AL for combating the *E. Coli* and *S. Aureus* biofilms.

In this work, a Ce‐doped lignin‐based hydrolase nanozyme (Ce‐AL) was synthesized using aminated lignin as a green ligand (Scheme 1a). The Ce‐AL exhibited selective hydrolysis of phosphate substrates rather than carbonate substrates, with stronger activity toward polyphosphate substrates than monophosphate substrates. Moreover, Ce‐AL demonstrated hydrolytic activity even toward the more stable amide bonds in proteins. Strikingly, contrasting with natural enzymes that denature at high temperatures, Ce‐AL displayed pronounced heat‐activated behavior, with its activity at 100°C being 2.03 times that at 40°C. This unique property, coupled with its multifunctionality, enabled Ce‐AL to effectively eradicate robust biofilms of both Gram‐negative (*Escherichia coli*) and Gram‐positive (*Staphylococcus aureus*) bacteria (Scheme 1b). This work not only achieves the valorization of industrial lignin into a high‐value functional material, but also expands the nanozyme toolbox with a sustainable and heat‐activated hydrolase nanozyme, demonstrating the versatility of lignin beyond oxidoreductase‐mimicking systems.

## 2 Results and Discussion

### 2.1 Synthesis and Characterization of Ce‐doped Lignin‐Based Hydrolase Nanozyme (Ce‐AL)

**FIGURE 1:**
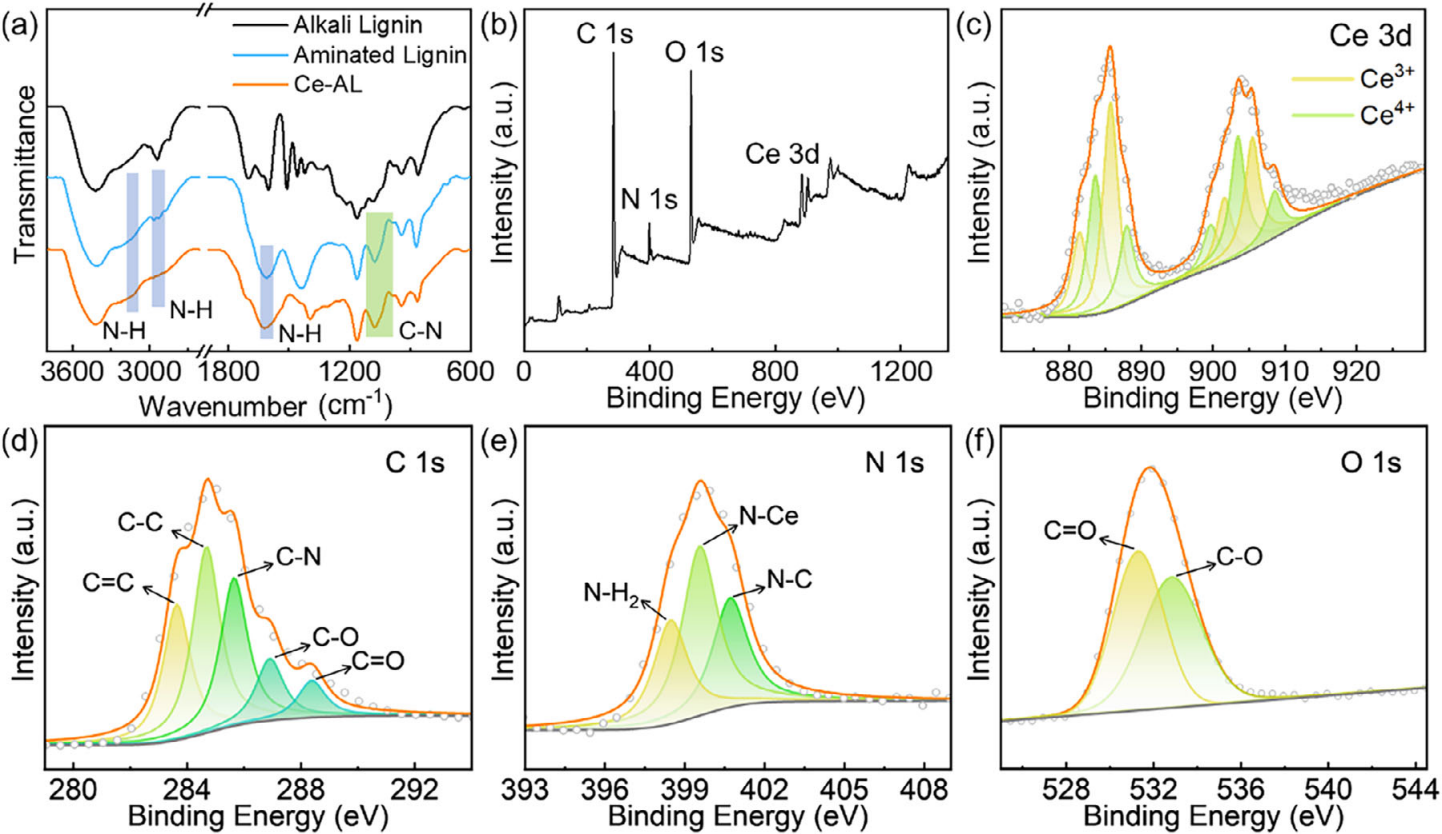
(a) FT‐IR spectra of alkali lignin, aminated lignin, and Ce‐AL; (b) XPS survey scan and high‐resolution spectra of (c) Ce, (d) C, (e) N, and (f) O elements of Ce‐AL.

The preparation process of Ce‐AL is illustrated in Scheme 1a, and detailed procedures were described in Section 4.1. As shown in the SEM and SEM‐EDS images (Figure S1), Ce‐AL was irregularly‐shaped nanoparticles with uniformly distributed C, N, O, and Ce elements. The XRD spectra (Figure S2) showed only broad diffraction peaks near 2*θ* = 22.5° and 42.8° of highly disordered carbon atoms, indicating that Ce was present in the form of coordination compounds rather than oxides. This conclusion was further supported by the blue shift of N–H (2910 cm^−1^) and C–N (1620 cm^−1^) peaks in the FT‐IR spectrum of Ce‐AL compared to aminated lignin (Figure 1a), as well as by the characteristic peaks of Ce 3d in the XPS spectrum at 886 and 905 eV (Figure 1b). The XPS quantification (Figure 1c) revealed that Ce in Ce‐AL existed in both trivalent (51.39%) and tetravalent (48.61%) states. The high‐resolution XPS spectra of C, N, and O elements (Figure 1d–f) confirmed that the characteristic chemical bonds from the aminated lignin precursor (e.g., C═C, C─N, C─O, C═O, and N─H_2_) were well preserved in Ce‐AL. Crucially, deconvolution of the N 1s spectrum provided direct evidence for the formation of the proposed Ce‐N active sites. A new peak emerged at 399.2 eV in Ce‐AL (Figure 1e), which was absent in the spectrum of the aminated lignin precursor (Figure S3). This peak is uniquely assigned to Ce‐N coordination. This binding energy was in excellent agreement with values reported for atomically dispersed Ce‐N_x_ centers in other catalytic materials, providing direct spectroscopic evidence for the successful coordination of Ce atoms to nitrogen within the lignin matrix [28].

### 2.2 The Catalytic Activity of Ce‐AL

**FIGURE 2:**
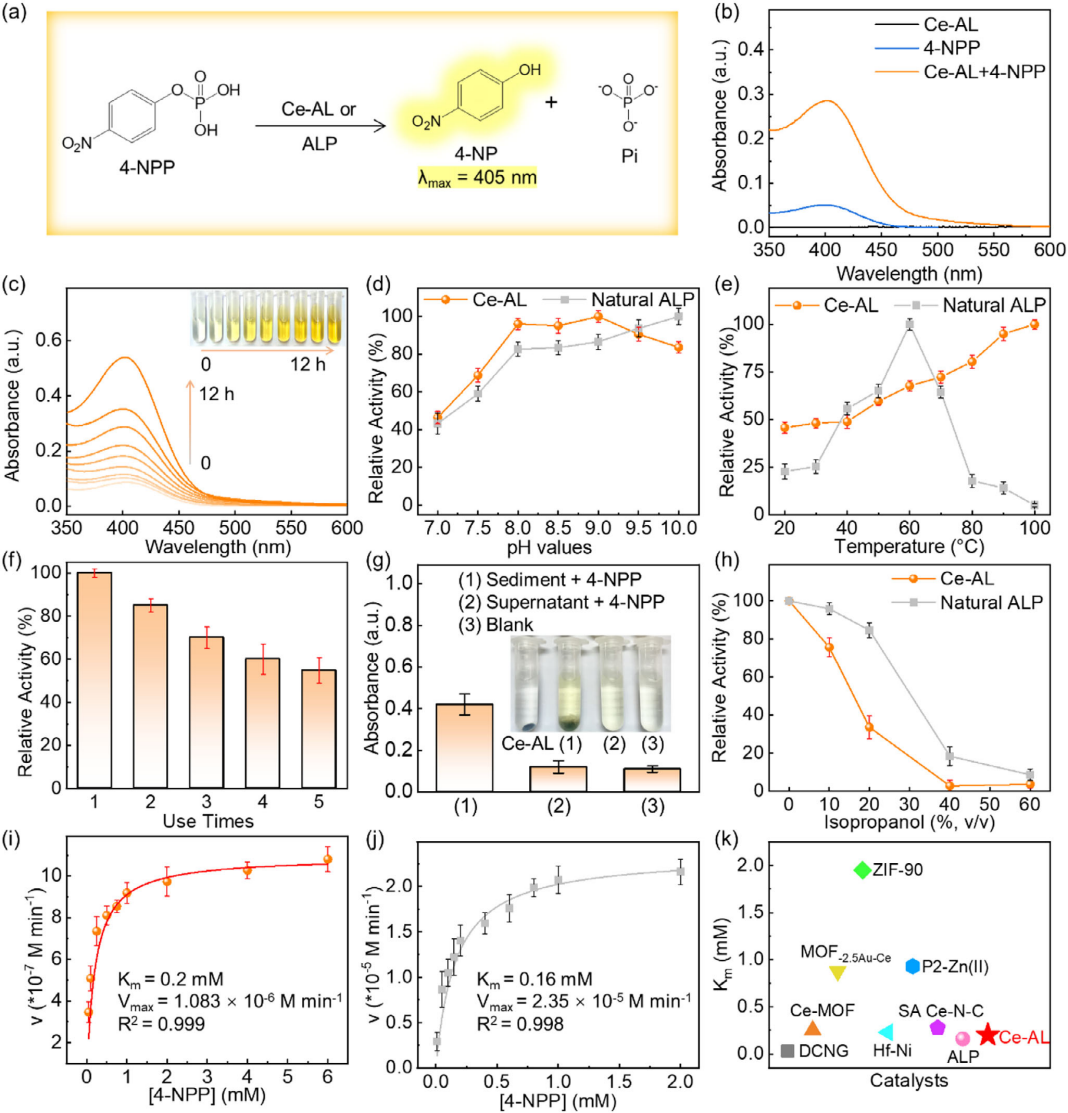
(a) Hydrolysis reaction of 4‐NPP; (b) UV–vis spectra of reaction systems; Effects of (c) time, (d) pH, (e) temperature, and (f) recycling on hydrolysis; (g) Absorbance at 405 nm of (1) sediment, (2) supernatant, and (3) blank control; (h) Effect of isopropanol on relative activity of Ce‐AL and ALP; Michaelis‐Menten fitting curves for (i) Ce‐AL and (j) ALP; (k) Comparison of *K_m_* values among various catalysts on 4‐NPP hydrolysis [12, 41, 42, 43, 44, 45, 46]. Error bars represent the standard deviation from five independent experiments.

The phosphatase‐like activity of Ce‐AL was initially investigated, as the cleavage of phosphate ester bonds represents a fundamental biochemical reactions [29]. As shown by the colorimetric reaction in Figure 2a, both Ce‐AL and alkaline phosphatase (ALP) hydrolyze *p*‐nitrophenyl phosphate (4‐NPP) to produce yellow *p*‐nitrobenzene (4‐NP, λ_max_ = 405 nm) and inorganic phosphate (Pi) under alkaline conditions. Evidently, Ce‐AL significantly accelerated the hydrolysis of 4‐NPP, demonstrating its intrinsic phosphate‐like activity (Figure 2b).

Furthermore, the synthesis of Ce‐AL was systematically optimized by modulating the concentration of the cerium precursor (Figure S4, cerium contents listed in Table S1). As the dosage of the cerium precursor increased during doping, the Ce content of Ce‐AL rose, which enhanced the relative catalytic activity of Ce‐AL. Based on Ce‐doping efficiency, Ce‐AL‐1.0 was selected for subsequent experiments. The yield of 4‐NP increased with higher dosages of Ce‐AL (Figure S5) and longer reaction time (Figure 2c). For optimal catalytic efficiency, a concentration of 0.5 mg mL^−1^ Ce‐AL was used in further experiments. Similar to ALP, Ce‐AL also exhibited maximal activity under weak alkaline conditions (pH 8.0–9.0, Figure 2d).

Notably, Ce‐AL showed a pronounced heat‐activated behavior, for its hydrolase‐like activity at 100°C was 2.03 times higher than that at 40°C (Figure 2e). To gain deeper mechanistic insight into this unique property, in situ diffuse reflectance infrared spectroscopy was performed across a temperature range of 20°C to 120°C (experimental details are provided in the Supporting Information). For the aminated lignin ligand alone, increasing temperature sharpened the O─H/N─H stretching bands (3530 and 3386 cm^−1^) and intensified spectral fluctuations in the 1250–1700 cm^−1^ region, indicating enhanced thermal motion and structural flexibility. In contrast, Ce‐AL displayed significantly suppressed spectral fluctuations in the 1250–1700 cm^−1^ region, demonstrating that the Ce‐N_x_ coordination structure effectively constrains the thermal disorder of the lignin scaffold. Furthermore, thermal‐induced shifts were observed for Ce‐AL: bands at 1280 and 1030 cm^−1^ shifted to lower wavenumbers, while the peak at 880 cm^−1^ shifted to a higher wavenumber. These shifts suggested a thermal‐tuning effect of the Ce‐N_x_ centers, potentially optimizing their local electronic structure and thereby enhancing their capability to polarize and activate substrates at elevated temperatures. Thus, the heat‐activated behavior of Ce‐AL originated not merely from accelerated mass transfer of substrate molecules, but also from the intrinsic thermal stability of the Ce‐N_x_ sites and the thermal optimization of their catalytic electronic structure.

The reusability of Ce‐AL was found to be limited, with an activity drop observed after the first cycle (Figure 2f). To elucidate the origin of this deactivation, the spent catalyst (denoted as Ce‐AL‐U1) was thoroughly characterized. XPS survey scans revealed the emergence of phosphorus and a significantly enhanced oxygen signal in Ce‐AL‐U1 (Figure S7a). High‐resolution P 2p spectra showed doublet peaks at 132.10 eV (P 2p_3/2_) and 133.04 eV (P 2p_1/2_), and the O 1s spectrum exhibited a new and intense peak at ∼530 eV (Figure S7b,c), collectively indicating the adsorption and formation of phosphate‐containing species on the catalyst surface [30, 31]. Compared to fresh Ce‐AL (Ce^3+^: 51.39%; Ce^4+^: 48.61%), the relative content of Ce^3+^ in Ce‐AL‐U1 decreased (Ce^3+^: 49.49%; Ce^4+^: 50.51%), indicating electron transfer associated with Pi coordination [32]. Furthermore, the N 1s spectrum of Ce‐AL‐U1 showed a weakened Ce‐N peak at 399.2 eV and the appearance of a new peak at 405.0 eV (Figure S7d), indicating charge redistribution on N atoms adjacent to Ce centers upon Pi binding. Correspondingly, the C 1s spectrum showed an increased C═O intensity, demonstrating electron transfer from the lignin aromatic structure during the process (Figure S7e). FT‐IR analysis further confirmed these findings (Figure S8): the intensity of the N─H band at 1620 cm^−1^ increased, and new peaks emerged at 881 and 1084 cm^−1^, assigned to P─O and P═O stretching vibrations, respectively [30]. These spectroscopic signatures proved a strong interaction between the Lewis acidic Ce sites and the Lewis basic phosphate product (Pi). The ICP‐OES measurement confirmed that the Ce content in Ce‐AL‐U1 remained at 9.726 wt.% with a concomitant P content of 1.19 wt.%, which indicated retention of the Ce‐N_x_ centers and the formation of Ce‐Pi complexes, rather than active site leaching. This phenomenon of phosphate product inhibition was consistent with that observed in CeO_2_ nanoparticles, confirming that the hydrolysis of 4‐NPP by Ce‐AL is a self‐limiting reaction [33].

To establish the phosphatase specificity of Ce‐AL, potential interference from other enzyme‐like activities was evaluated and ruled out. As shown in Figure S9a–d, Ce‐AL showed no detectable peroxidase‐, oxidase‐, catalase‐, and laccase‐like activities. Although weak superoxide dismutase‐like activity was observed under neutral conditions (Figure S9e), the hydrolysis of 4‐NPP under alkaline conditions does not involve radical species [34], confirming that this activity does not interfere with the phosphatase assay.

Further assays were performed to confirm the catalytic origin of 4‐NPP hydrolysis. The Ce‐AL solution (1 mg mL^−1^) was separated into sediment and supernatant fractions. Only the sediment‐contained system exhibited significant absorbance corresponding to the hydrolysis products of 4‐NPP (Figure 2g), indicating that the catalytic activity originated from the solid Ce‐AL nanoparticles rather than leached Ce ions. Given that water ionization is essential for natural ALP catalysis [35], the impact of isopropanol (a competitive nucleophilic cosolvent) on the activities of both Ce‐AL and ALP was compared (Figure 2h). The relative activity of Ce‐AL and ALP both decreased gradually with increasing isopropanol proportion, indicating that water molecules participated in the Ce‐AL‐catalyzed 4‐NPP hydrolysis reaction.

As revealed by XPS, the ratio of Ce^3+^ to Ce^4+^ was near 1:1. In conventional ceria nanomaterials, such a mixed‐valence state is a hallmark of redox enzyme‐like activities (e.g., oxidase, catalase) [36, 37]. However, Ce‐AL showed no significant redox activity (Figure S9). We proposed that the Ce^3+^/Ce^4+^ pair synergistically constructed and optimized potent Lewis acid sites, which are pivotal for hydrolysis. The Ce^4+^ ions in Ce‐AL functioned as strong Lewis acids centers, polarizing and activating the electrophilic phosphorus atom in phosphate ester substrates [38]. Simultaneously, the presence of Ce^3+^ often associated with coordinatively unsaturated environments, promoted the adsorption and activation of nucleophilic water molecules [39, 40]. This synergistic effect between Ce^3+^and Ce^4+^ created a highly effective Lewis acid‐base catalytic center for hydrolyzing the phosphate ester bonds. This proposed mechanism is consistent with our experimental evidence: (1) the lack of prominent redox enzyme‐like activities demonstrated that the mixed valence was not primarily engaged in electron transfer (Figure S9), and (2) the hydrolysis reaction relied on water as a nucleophile (Figure 2h).

### 2.3 Steady‐State Kinetic Analysis of Ce‐AL

The steady‐state kinetic of 4‐NPP hydrolysis catalyzed by Ce‐AL and ALP were evaluated using the Michaelis‐Menten model (Figure 2i,j). The *K_m_* value of Ce‐AL toward 4‐NPP was determined to be 0.2 mm, which is close to that of ALP (0.16 mm). The *K_cat_* of Ce‐AL was 0.001 s^−1^ (Table S2), indicating potential for improvement in catalytic efficiency. This may be attributed to the heterogeneous nature of the lignin scaffold where not all Ce‐N_x_ active sites were uniformly accessible.

The superior substrate affinity (*K_m_* = 0.20 mm) of Ce‐AL was not only comparable to that of natural ALP, but also ranked favorably among many advanced hydrolytic nanozymes, including those based on metal–organic frameworks or noble metals (Figure 2k; Table S2) [12, 41, 42, 43, 44, 45, 46]. More significantly, Ce‐AL achieved this high affinity coupled with unique heat‐activated behavior using industrial lignin as an economical and renewable platform, thereby establishing a novel route for the valorization of biomass into high‐value functional nanomaterials.

Future work to enhance the catalytic turnover frequency will focus on engineering the Ce coordination microenvironment (e.g., by adjusting N‐doping and local electronic structure) and optimizing the porosity of the lignin‐derived scaffold to facilitate substrate diffusion and active site utilization [47, 48, 49].

### 2.4 Substrate Diversity of Ce‐AL

**FIGURE 3:**
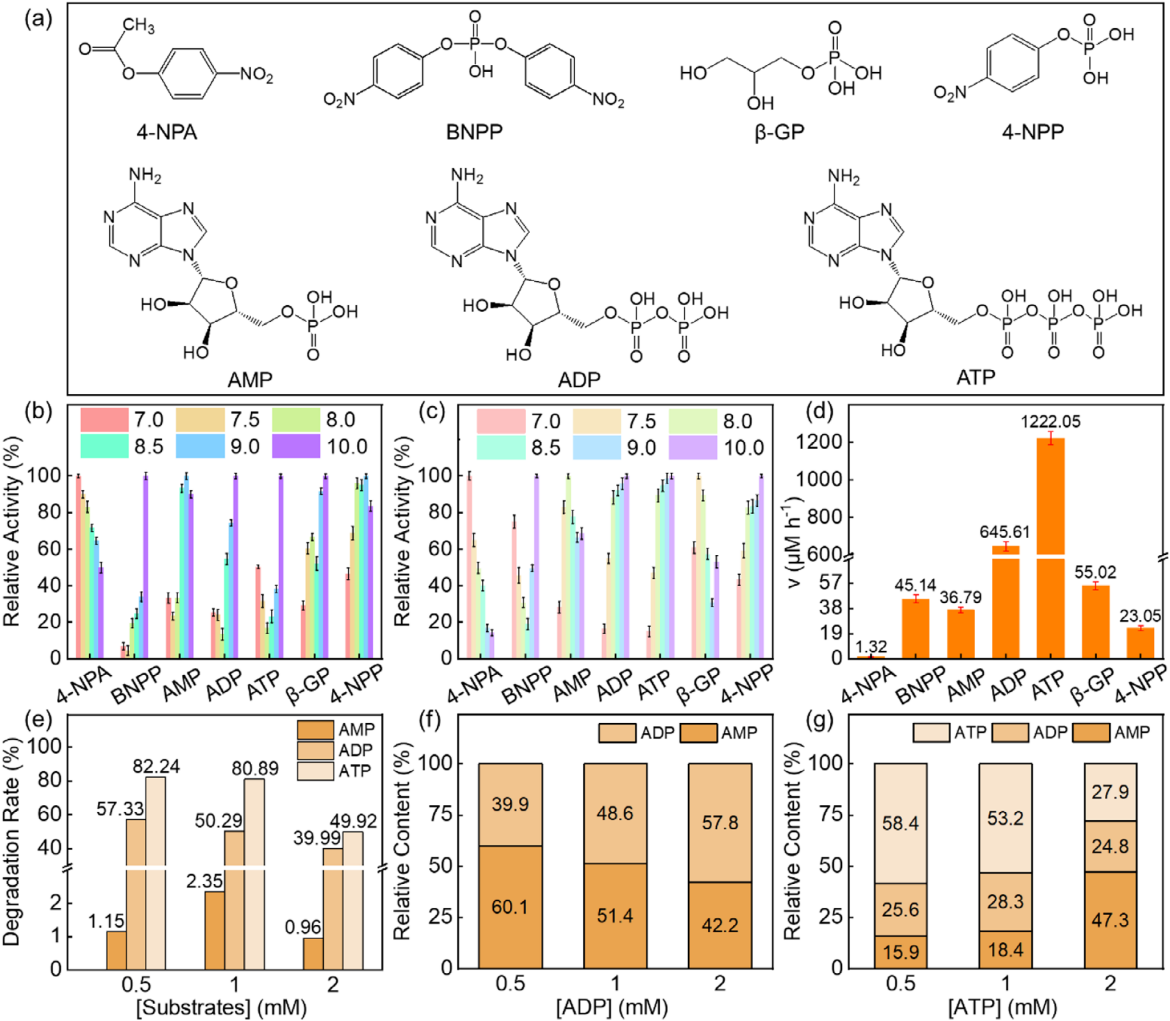
(a) Chemical structures of different substrates; Effect of pH values on different substrates hydrolysis catalyzed by (b) Ce‐AL and (c) ALP; (d) Hydrolysis rates of different substrates by Ce‐AL; (e) Degradation rates of AMP, ADP, and ATP at different concentrations by Ce‐AL; Relative content of each component in the hydrolysis products of (f) ADP and (g) ATP catalyzed by Ce‐AL. Error bars represent the standard deviation from five independent experiments.

To explore the catalytic versatility of Ce‐AL, its hydrolytic activity toward seven phosphate substrates were evaluated (Figure 3a). The optimal pH values differed between Ce‐AL and ALP depending on the substrates (Figure 3b,c; Table S3). These substrate‐specific pH conditions were maintained in all subsequent assays. The hydrolysis rates of different substrates after 1 h were compared (Figure 3d). Among them, 4‐nitrophenyl acetate (4‐NPA) remained virtually unhydrolyzed, whereas both mono‐ and poly‐phosphate substrates were cleaved, demonstrating Ce‐AL's selectivity toward organic phosphate esters. The reaction equations for 4‐NPP, bis(4‐nitrophenyl) phosphate (BNPP), and β‐glycerophosphate (β‐GP) are provided in Figure S10a,b.

The distinct selectivity of Ce‐AL toward phosphate esters over carbonate ester 4‐NPA could be rationalized by considering both electronic and structural factors. According to the hard‐soft acid‐base theory, the Ce^4+^ site in Ce‐AL acted as a strong Lewis acid and preferentially coordinate with phosphate esters, which exhibited stronger Lewis basicity than carbonate esters [13]. Moreover, the tetrahedral geometry and different electronic structure of the phosphate group likely facilitate a more favorable coordination geometry with the Ce‐N_x_ active center, whereas the planar structure of the carbonate ester may lead to steric hindrance or electronic incompatibility. This substrate specificity underscored the enzyme‐like selectivity of Ce‐AL, which was crucial for the targeted degradation of specific biomolecules, rather than non‐specific hydrolysis.

For equimolar concentrations of adenine monophosphate (AMP), adenosine‐diphosphate (ADP), adenosine triphosphate (ATP), Ce‐AL exhibited the following degradation rate trend: ATP > ADP >> AMP (Figure 3d,e), resembling natural apyrase [50] and apyrase‐like CeO_2_ nanozyme [51]. This trend might be attributed to the higher stability of the phosphate bond in AMP and steric hindrance limiting access to the catalytic center (Figure S10c).

In ADP hydrolysis system, as the ADP concentration increased, the relative content of AMP decreased (Figure 3f), suggesting that AMP may become the primary substrate once its relative content exceeded that of ADP during hydrolysis. Thus, Ce‐AL catalyzed ADP hydrolysis proceeded in two steps: initial hydrolysis of ADP into AMP and Pi, followed by AMP slow hydrolysis (Figure S10d).

For the hydrolysis of ATP, ADP, and AMP were detected simultaneously across all ATP concentrations tested (Figure 3g). When the concentrations of ATP were 0.5 and 1 mm, ADP was the dominant product. At 2 mm ATP, the decrease in ATP level shifted the primary substrate to ADP, accompanied by AMP accumulation, suggesting multi‐step hydrolysis due to substrate competition (Figure S10e).

Peptide bonds in proteins exhibit exceptional stability, with an autolysis half‐life of approximately 350 years under physiological pH and temperature [52]. To assess the proteolytic activity of Ce‐AL, bovine serum albumin (BSA), a globular protein comprising 585 amino acids, was selected as a model substrate due to its structural stability and broad relevance. Sodium dodecyl sulfate‐polyacrylamide gel electrophoresis (SDS‐PAGE) analysis (Figure S11) of the hydrolysis products of BSA revealed that ALP showed almost no hydrolytic effect on BSA for its inherent specificity, whereas the Ce‐AL could hydrolyze BSA partially. Notably, Ce‐AL exhibited enhanced hydrolysis activity toward BSA in Tris‐HCl buffer at pH 8.0.

### 2.5 Bacterial Biofilm Removal by Ce‐AL

**FIGURE 4:**
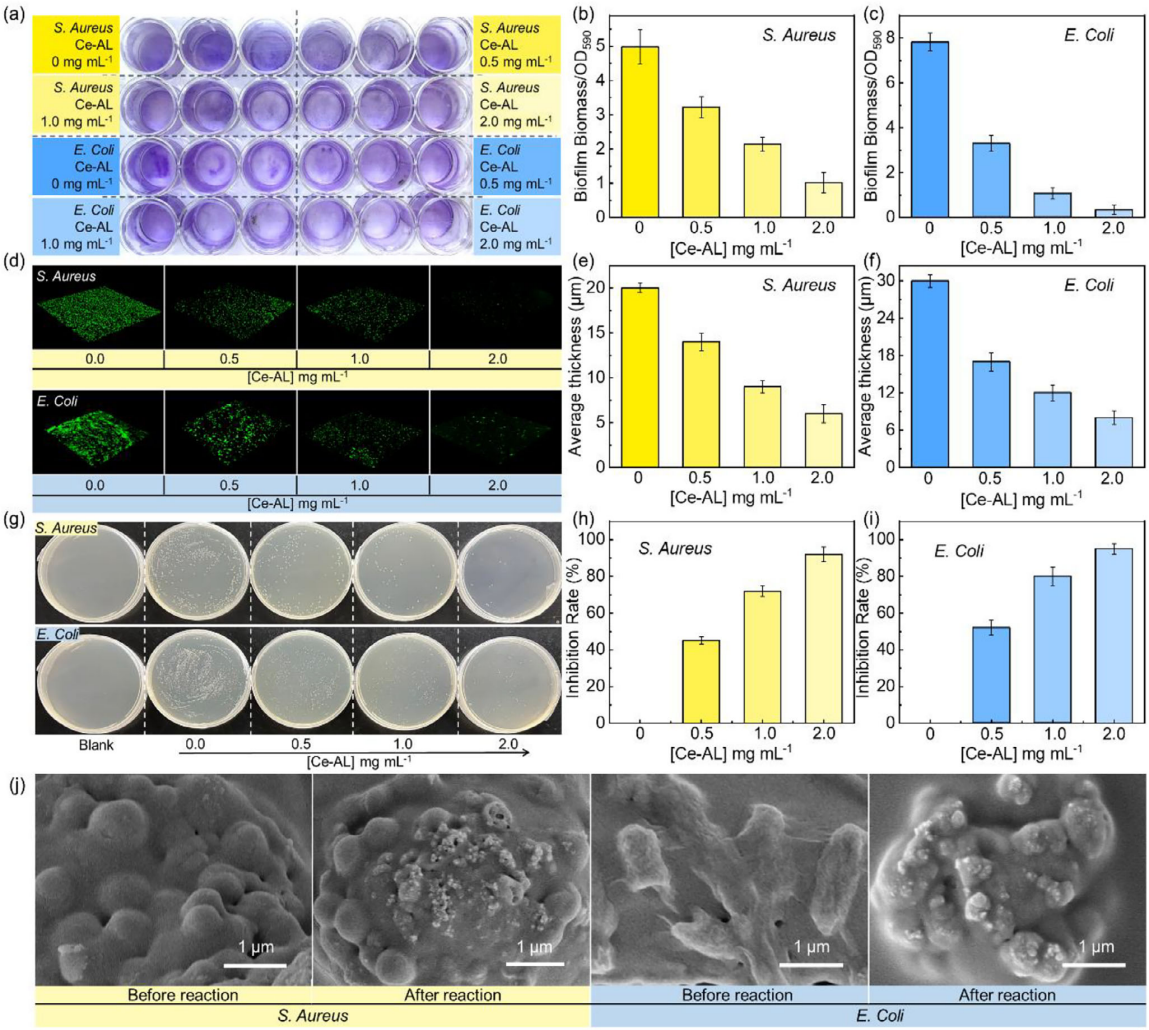
(a) Optical images and (b,c) total biomass quantified by crystal violet staining in S. *Aureus*, and *E. coli* biofilms after treatment with different concentrations of Ce‐AL; (d) CLSM fluorescence images of FDA‐stained biofilms after 24 h of Ce‐AL treatment at 37°C (green = live cells); (e f) Corresponding biofilm thickness profiles; (g) Optical images of colony formation after 24 h of biofilms treatment at 37°C with different concentrations of Ce‐AL observed via plate counting; (h,i) Corresponding biofilms inhibition rates; (j) SEM images of biofilms before and after treatment with 2.0 mg mL^−1^ Ce‐AL at 37°C for 24 h. Error bars represent the standard deviation from five independent experiments.

Biofilms are multicellular communities formed by bacteria and their self‐synthesized extracellular polymeric substances (EPS) matrix [9]. The EPS is primarily composed of polysaccharides, proteins, and nucleic acids, which collectively form a physicochemical barrier that confers resistance to antibiotics, immune clearance, and environmental stressors [53]. The robustness of biofilms makes their eradication particularly challenging. Ce‐AL efficiently hydrolyzed key EPS components (e.g., structural proteins and extracellular ATP) at the physiologically relevant temperature of 37°C. Notably, it further exhibited a heat‐activated behavior, with significantly enhanced activity at elevated temperatures (> 60°C). The total biomass of *S. aureus* and *E. coli* biofilms after Ce‐AL treatment was quantified by crystal violet staining. As the concentration of Ce‐AL increased, the stained biofilms exhibited a lighter purple color (Figure 4a). Correspondingly, the biomass decreased significantly following Ce‐AL treatment (Figure 4b,c), demonstrating the effective hydrolysis of EPS components.

Biofilms of *S. aureus and E. coli* were stained with fluorescein diacetate (FDA) and imaged by confocal laser scanning microscopy (CLSM) before and after Ce‐AL treatment (Figure 4d). Fluorescence intensity was markedly reduced after Ce‐AL treatment, and the thickness of the biofilms decreased with increasing Ce‐AL concentration (Figure 4e,f). Furthermore, the plate counting method was used to evaluate the biofilm inhibition after 24 h of Ce‐AL treatment. The number of colonies decreased progressively with higher Ce‐AL concentrations (Figure 4g). Notably, 2.0 mg mL^−1^ Ce‐AL achieved inhibition rates of 92.3% for *S. aureus* and 95.1% for *E. coli* biofilms, separately (Figure 4h,i). Scanning electron microscopy (SEM) images revealed that untreated samples contained characteristic spherical (*S. aureus*) and rod‐shaped (*E. coli*) cells embedded within a dense extracellular matrix (Figure 4j). After Ce‐AL treatment, visible pores and disruptions appeared on the bacterial cell walls, and the connections between surface bacteria were also damaged.

The anti‐biofilm mechanism of Ce‐AL involved a dual‐targeting mechanism. On the one hand, substrate selectivity assays revealed that Ce‐AL efficiently hydrolyzes key proteinaceous and nucleotide components of the EPS, while exhibiting negligible activity toward model polysaccharides such as alginate (Figure S12). This selective hydrolysis effectively dismantled the EPS framework by targeting specific structural and functional biopolymers, rather than through nonspecific matrix degradation. On the other hand, direct morphological evidence from SEM imaging (Figure 4j) demonstrated that Ce‐AL inflicts substantial physical damage to bacterial cell walls. Therefore, Ce‐AL combated biofilms via a concerted two‐pronged mechanism: (1) selectively degrading critical EPS components to disrupt the protective biofilm architecture, and (2) directly attacking and lysing the embedded bacterial cells.

## 3 Conclusions

In this work, a Ce‐doped lignin‐based hydrolase‐like nanozyme (Ce‐AL) was successfully synthesized using industrial alkali lignin precursor. The Ce‐AL selectively hydrolyzed the phosphate ester bonds (including monophosphate and polyphosphate substrates) over carbonate ester bonds, and even cleaved stable proteins amide bonds. It demonstrated a high substrate affinity (*K_m_* = 0.20 mm) comparable to natural alkaline phosphatase and a unique heat‐activated behavior, with activity at 100°C being 2.03‐fold that at 40°C, due to the synergistic effect of enhanced mass transfer and thermal optimization of the Ce‐N_x_ centers. Leveraging these properties, Ce‐AL achieved over 90% inhibition of both *S. Aureus* and *E. coli* biofilms at a concentration of 2.0 mg mL^−1^. by selectively degrading key EPS components (proteins and nucleotides) and directly damaging bacterial cells. This work validates lignin as a versatile platform for fabricating sustainable and stimuli‐responsive nanozymes, promoting the high‐value valorization of biomass.

## 4 Materials and Methods

### 4.1 Materials

Longlive Bio‐Technology Co, Ltd. (Shandong, China) provided the industrial alkali lignin (extracted from corn cob, hydroxyl content > 3.0%, water content < 14.0%, ash content < 5.0%, weight‐average molecular weight: 2270 g mol^−1^, number‐average molecular weight: 1490 g mol^−1^, polydispersity: 1.52, β‐O‐4 content: 24.05%), which was used as received without further purification or fractionation. The epichlorohydrin, triethylenetetramine, sodium hydroxide, ethanol, N, N‐dimethylformamide, ceric ammonium nitrate, crystal violet, 4‐nitrophenyl acetate, bis(4‐nitrophenyl) phosphate, 4‐nitrophenyl phosphate were supplied by Macklin Biochemical Co., Ltd (Shanghai, China). Alkaline phosphatase (from calf intestine, ammonium sulfate suspension, 4000 U mg^−1^) and fluorescein diacetate were purchased from Yuanye Biotechnology Co., Ltd. (Shanghai, China). Deionized water was used in all experiments. Bovine serum albumin (BSA, from bovine serum, ∼66.5 kDa) was purchased from Sigma‐Aldrich Chemical Reagent Co., Ltd. *Escherichia coli* (*E*. coli, K‐12 strain, ATCC 10798) and *Staphylococcus aureus* (*S*. aureus, ATCC 25923) were acquired from the China General Microbiological Collection Center.

### 4.2 Synthesis of the CeN_x_‐Centered Lignin‐based Nanozymes (Ce‐AL)

First, the lignin‐based ligand was synthesized according to our previous work [27]. The obtained lignin‐based ligand (0.25 g) and cerium sources (ceric ammonium nitrate, 0.25, 0.50, 1.00, 1.50 g) were dispersed in N, N‐dimethylformamide (30 mL) and kept at 110°C for 10 h. After removing free ceric ions and residual organic solvent, the Ce‐AL was obtained.

To ensure reproducible synthesis of Ce‐AL from the inherently heterogeneous industrial lignin, a standardized chemical functionalization approach was employed. The raw lignin was first subjected to a controlled amination reaction. The process converted lignin into a ligand with a more consistent distribution of amine and alcohol hydroxyl groups, thereby providing a uniform coordination environment for Ce ions and mitigating the initial variability in molecular weight and subunit (S/G) composition of the feedstock. Subsequent coordination with Ce under. Subsequent coordination with Ce under strictly controlled conditions (solvent, time, temperature, and pH) ensured the reliable formation of the target Ce‐N_x_ active sites.

This approach of engineering lignin into a robust ligand platform for constructing well‐defined catalysts were well‐established, as demonstrated in previous works on metal‐lignin coordination [54, 55].

### 4.3 Characterizations

Hitachi 3500S scanning electron microscopy (SEM) and energy dispersive spectrometer (SEM‐EDS) were used for the morphological and elemental distribution observation, respectively. Using the Nicolet iN10 FT‐IR spectrometer at 25°C to obtain the Fourier transform infrared (FT‐IR) spectra. The ESCALAB 250Xi X‐ray photoelectron spectroscopy (XPS) and Bruker D8 ADVANCE diffractometer (XRD) were used for element composition and crystal structure analysis in Ce‐AL samples. An Agilent 5110 inductively coupled plasma optical emission spectrometer (ICP‐OES) was used to measure the copper content in Ce‐AL samples.

### 4.4 Evaluations of the Catalytical Activities of Ce‐AL and Natural Alkaline Phosphatase

With a total volume of 3 mL, the peroxidase‐, oxidase‐, catalase‐, laccase‐, and superoxide dismutase‐like activities of Ce‐AL were evaluated. The details are listed in Table S4.

The yellow hydrolysis product 4‐nitrobenzene (4‐NP, λ_max_ = 405 nm, ε_405 nm_ = 18.5 m ^−1^ cm^−1^) of 4‐nitrophenyl phosphate (4‐NPP) was measured for evaluating the hydrolase like activity of Ce‐AL. In a typical issue, buffer solution (Tris‐HCl, pH 9.0, 20 mm) contained the Ce AL (1 mg mL^−1^) and 4‐NPP (2 mg mL^−1^, in DMSO) was incubated for 30 min. Before the absorbance measurement, a PES disposable filter (0.22 µm) was used to remove Ce‐AL from the solution system. The used Ce‐AL was washed with ethanol and collected for recycling experiments. As for the natural alkaline phosphatase (ALP), the 0.33 U mL^−1^ of ALP was used instead of lignin‐based nanozymes. and the mixed solution was incubated at 37°C for 60 min. The diethanolamine‐HCl buffer (pH 10.0, 1 m, containing 1 mm MgCl_2_) was used. The catalytic activity of Ce‐AL and ALP of 20–100 °C and pH 7–10 was tested to investigate the optimal conditions.

### 4.5 Steady‐State Kinetics Analysis of Ce‐AL and ALP

The steady‐state kinetics of Ce‐AL and ALP were measured using the hydrolysis reaction of 4‐NPP. The concentration of 4‐NPP was 1–6 mm, and the final concentration of Ce AL was 1 mg mL^−1^. The calculation equation of apparent kinetic parameters was calculated as Equation (1):

(1) $$v=v_{max}\times \left[S\right]/\left(k_{m}+\left[S\right]\right)\;$$

where *v* (m s^−1^) and v_max_ (m min^−1^) correspond to the initial velocity and the maximal reaction velocity, respectively, [*S*] (m) represents the concentration of substrate, and *K_m_* (m) means the Michaelis‐Menten constant.

The catalytic constant (*K_cat_*) was calculated as Equation (2):

(2) $$K_{cat}=v_{max}/E\;$$

where *E* (m) means the concentration of cerium activity center of Ce‐AL in the colorimetric system (detected by ICP‐OES).

### 4.6 Hydrolysis of Various Substrate Using Ce‐AL

For the Ce‐AL (0.5 mg mL^−1^) hydrolyzed nitrophenyl acetate (4‐NPA) and *bis* (*p*‐nitrophenyl) phosphate (BNPP) (final concentration of 2 mm), the produced 4‐NP was quantified by the UV–vis spectrophotometry. For the hydrolysis of the 5'‐adenosine monophosphate (AMP), adenosine‐5'‐diphosphate (ADP), 5'‐adenosine triphosphate (ATP), β‐glycerophosphate (β‐GP) (final concentration of 2 mm) catalyzed by Ce‐AL (0.5 mg mL^−1^), the produced inorganic phosphate (Pi) was quantified using the molybdenum blue method (Figure S13). Furthermore, the hydrolysis products of AMP, ADP, and ATP were analyzed using liquid chromatography (LC). The products were quantified against standard curves (Figure S14), and the experimental details are shown in the Supporting Information. To observe the hydrolysis of amide bonds in BSA (1.0 mg mL^−1^), the Ce‐AL (2.0 mg mL^−1^) was used. After incubating in the phosphate buffer (PB) or Tris‐HCl buffer (50 mm, pH 7.5–8.0) for 12 h at 60°C, the mixed solution was filtered and the supernatant was analyzed by sodium dodecyl sulfate‐polyacrylamide gel electrophoresis (SDS‐PAGE).

### 4.7 Observation of the Hydrolysis Effect of Ce‐AL on Bacterial Biofilms

First, to obtain the mature bacterial biofilms of Gram‐negative bacteria (*E. coli*) and Gram‐positive bacteria (*S. aureus*), the bacterial suspensions (OD 0.01) were added onto cover glasses in a 24 well plate (with 1 mL of LB culture medium) and were incubated at 37°C for 24 h. After removing the LB culture medium, the Ce‐AL (0, 0.5, 1.0, 2.0 mg mL^−1^, in 1 mL of LB culture medium) was added to the 24‐well plate. The mixed solution was incubated at 37°C for 24 h, then the characterizations of crystal violet staining, laser confocal microscopy (CLSM), SEM, and antibacterial rate were carried out (details in Supporting Information).

### 4.8 Statistical Analysis

The quantitative data were expressed as the mean ± standard deviation (SD) from five independent experiments (*n* = 5). The error bars in the figures represented the SD, indicating the variability among the independent experimental replicates.

## Author Contributions

Xin Liu and Lijun Li contributed equally to this work, they performed conceptualization, Writing – original draft. Xue Zhang: Data curation, Formal analysis; Alireza Ashori: Software, Visualization; Fubao Sun: Funding acquisition, Methodology; Feng Xu: Project administration, Resources; Xueming Zhang: Software, Supervision, writing – review & edited; Yao Chen: Validation, Visualization, writing – review & edited. The authors read and approved the final manuscript.

## Conflicts of Interest

The authors declare no conflicts of interest.

## Supporting information




**Supporting File**: advs73874‐sup‐0001‐SuppMat.docx.

## Data Availability

Data will be made available on request.
