# Supplementary material for: Ce‐Doped Lignin‐Based Nanozyme with Heat‐Activated Hydrolase Activity
Source: Adv Sci (Weinh). 2026 Jan 23;13(18):e19268. doi: 10.1002/advs.202519268 (PMC13042988; doi:10.1002/advs.202519268)
Supplement: Supplementary file 1 — Supporting File: advs73874‐sup‐0001‐SuppMat.docx. [file ADVS-13-e19268-s001.docx]

Supporting Information

**Ce-Doped Lignin-Based Nanozyme with Heat-Activated Hydrolase Activity**

*Xin Liu, Lijun Li, Xue Zhang, Alireza Ashori, Fubao Sun, Feng Xu, Xueming Zhang^*^, Yao Chen^*^*

Xin Liu, Lijun Li, Feng Xu, Xueming Zhang

Beijing Key Laboratory of Lignocellulosic Chemistry, Beijing Forestry University, Beijing, 100083, China.

E-mail: xm_zhang@bjfu.edu.cn. Tel. and Fax: +86-01062336189

Xin Liu, Yao Chen

State Key Laboratory of Biopharmaceutical Preparation and Delivery, Institute of Process Engineering, Chinese Academy of Sciences, Beijing 100190, China.

E-mail: chenyao@nankai.edu.cn

Lijun Li, Xue Zhang

China National Pulp and Paper Research Institute Co., Ltd., Beijing 100102, China.

Alireza Ashori

Department of Chemical Technologies, Iranian Research Organization for Science and Technology (IROST), Tehran, Iran

Fubao Sun

Key Laboratory of Industrial Biotechnology, Ministry of Education, School of Biotechnology, Jiangnan University, Wuxi 214122, China

Xueming Zhang

State Key Laboratory of Bio-based Fiber Materials, Zhejiang Sci-Tech University, Hangzhou, 310018, China; Email: xmzhang@zstu.edu.cn

Xin Liu and Lijun Li contributed equally to this work.

Data will be made available on request. There are no conflicts to declare.

**1. Experimental details：**

1.1 DRIFT spectroscopy measurement

*In situ* diffuse reflectance infrared (DRIFT) spectroscopy was performed using a Nicolet iS10 Spectrometer (Thermo Fisher, USA) and the spectra were recorded in the region 4000-400 cm^-1^ at a resolution of 4 cm^-1^. Potassium bromide (KBr) powder was used as a sample matrix, and the mass of sample to KBr ratio was 5:100. The tests were conducted under air conditions. To examine the vibration of Ce-AL in response to temperature, the DRIFT spectra of the samples were detected at temperature varied from 20 ºC to 120 ºC. The heating rate was 5 ºC min^-1^, and after holding at each test temperature for 10 min, scanned 64 times to collect spectra.

1.2 Using the liquid chromatography (LC) to measure the hydrolysis rate of AMP, ADP, and ATP

The liquid chromatography (LC) test was conducted using an Ultrasphere ODS EC 250 × 4.60 mm column (Beckman Instruments, USA) equipped with a Beckman 125 pump system. The peak values were detected and analyzed at 254 nm using a Gold 168 diode array detector. Continuous gradient elution method was used for high-performance liquid chromatography separation. The mobile phase A used was 0.05 m phosphate buffer solution, and the mobile phase B was composed of 100% acetonitrile. Ultrasonic instrument was used to drive away the bubbles in the two solutions. The elution procedures were as follows: 0 min with 100% A, 0% B; 2 min with 95% A, 5% B; 4 min with 80% A, 20% B; 5.3 min with 75% A, 25% B, and 6 min with 100% A, 0% B. Finally, the program took another minute to return to its initial state and stabilize. The flow rate of the mobile phase was 1.2 mL min^-1^, and the injection volume was 20 μL. The total retention time was about 5 min, and the gradient ran for 6 min to ensure complete separation. The ATP, ADP, and AMP in the samples were identified by comparing the retention time with the standard substances, and the concentrations of ATP, ADP, and AMP were determined with the external standard method. The data was represented as the average of six repeated measurements.

1.3 Crystal violet staining of bacteria biofilms

The biofilms were rinsed with physiological saline before been stained at room temperature for 30 min using crystal violet (1 mL, 1 g L^-1^). Then the biofilms were washed with physiological saline for three times to remove the residual dye, and their optical photos were taken. The crystal violet bound to the biofilm was removed by anhydrous ethanol and been diluted to an appropriate concentration for UV-vis measurement.

1.4 Evaluation of hydrolytic activity of Ce-AL toward sodium alginate

The hydrolytic activity of Ce‑AL toward sodium alginate (SA) was assessed by measuring the release of reducing sugars using the 3,5‑dinitrosalicylic acid (DNS) reagent.^[^[^1^](#_ENREF_1)^]^ SA (1 wt%) was incubated with Ce‑AL (1.0 mg·mL^-1^) in Tris‑HCl buffer (20 mM, pH 9.0) at 60 °C for 1 h. Then, DNS reagent (1 mL) was added, and the mixture was heated in a boiling water bath for 10 min. After cooling, the solution was filtered and diluted 10‑fold for the absorbance measurement at 540 nm. A blank control was performed by replacing SA with buffer. SA and glucose (1 mM) solutions were also measured as references.

1.5 Observation of bacteria biofilms using the laser confocal microscopy (CLSM)

The biofilms incubated with/without Ce-AL were stained using the fluorescein diacetate (FDA) dye at room temperature in the dark for 10 min before the CLSM observation with a 512 * 512 400Hz resolution.

1.6 Antibacterial rate of Ce-AL on bacterial biofilms using the plate coating counting method

The bacterial biofilms incubated with/without Ce-AL were rinsed in physiological saline and dispersed using ultrasonic treatment. After gradient dilution, the bacterial solutions were coated on LB solid medium for cultivated 37 °C for 12 h before the total numbers of colony were counted.

**2. Supporting figures：**


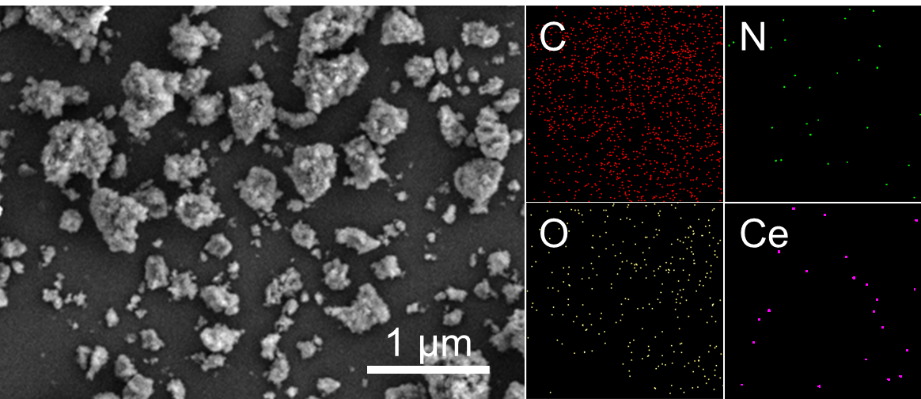


Figure S1. SEM and SEM-EDS images of Ce-AL

Figure S2. XRD diffraction pattern of Ce-AL.


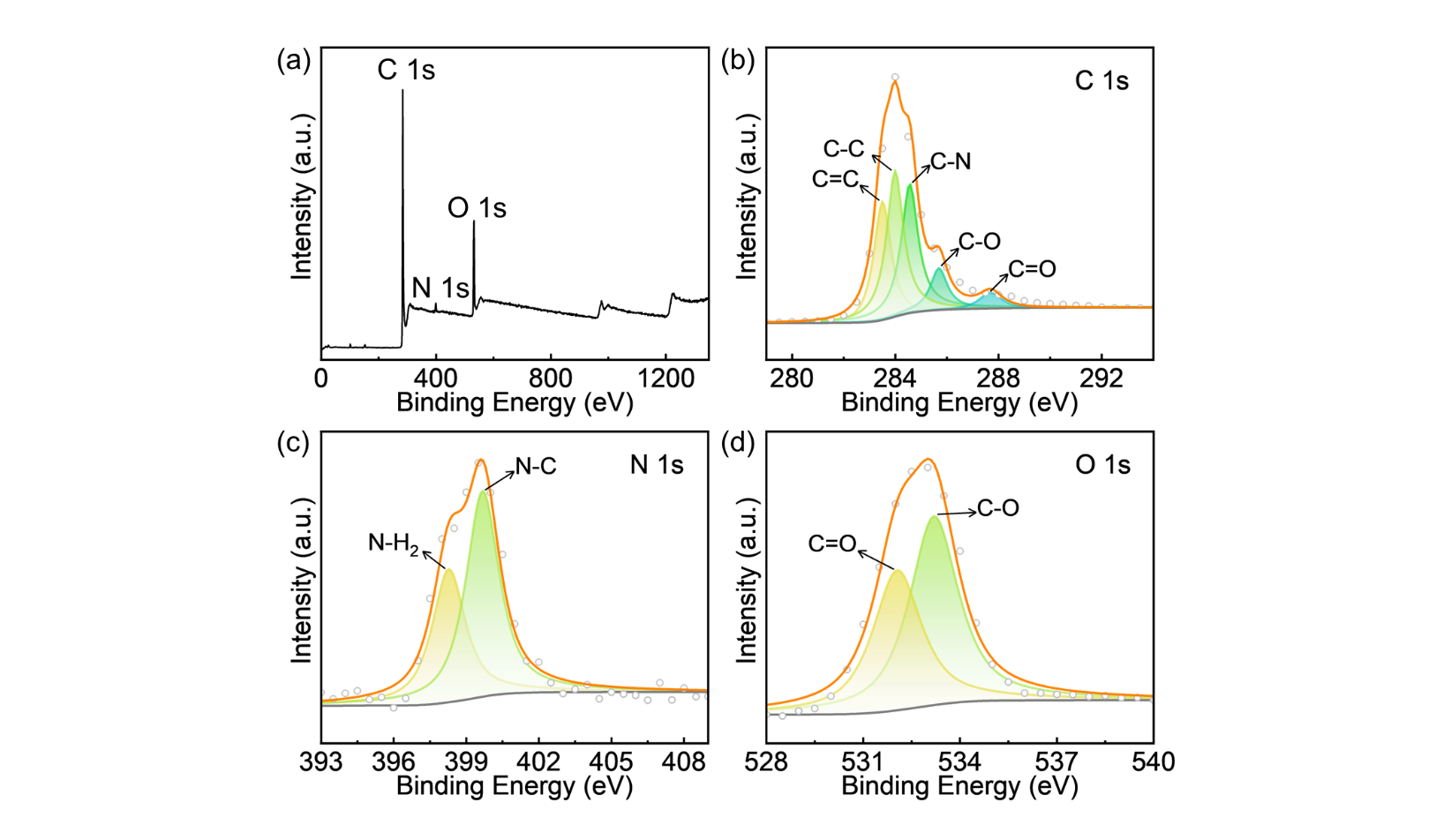


Figure S3. (a) XPS survey scan and high-resolution spectra of (b) C 1s, (c) N 1s, and (d) O 1s of aminated lignin.


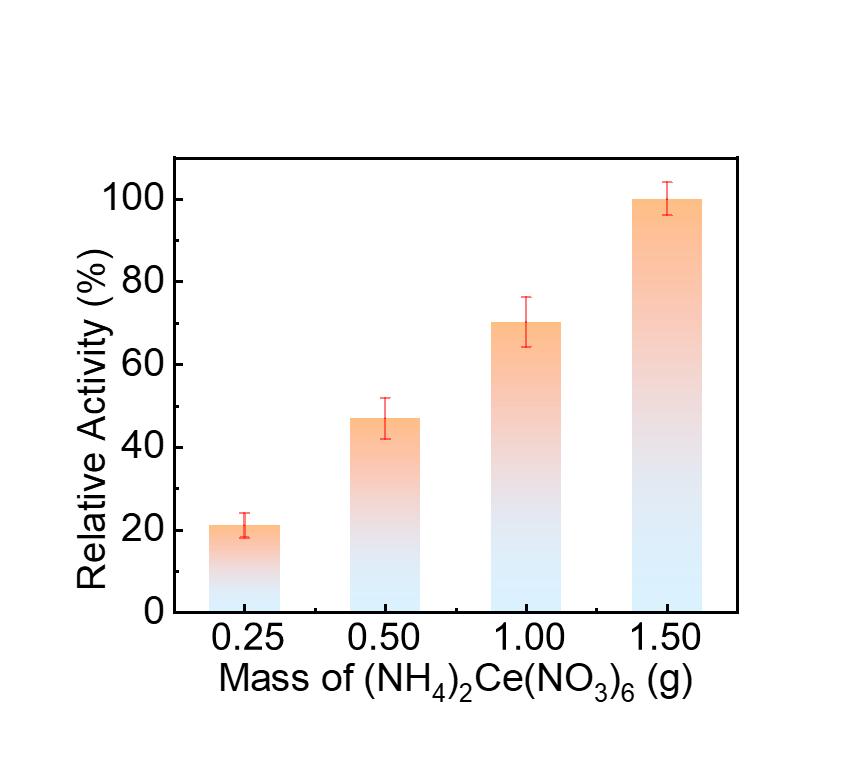


Figure S4. Relative hydrolase-like activity of Ce-ALs obtained from different qualities of cerium sources. Error bars represent the standard deviation from five independent experiments.


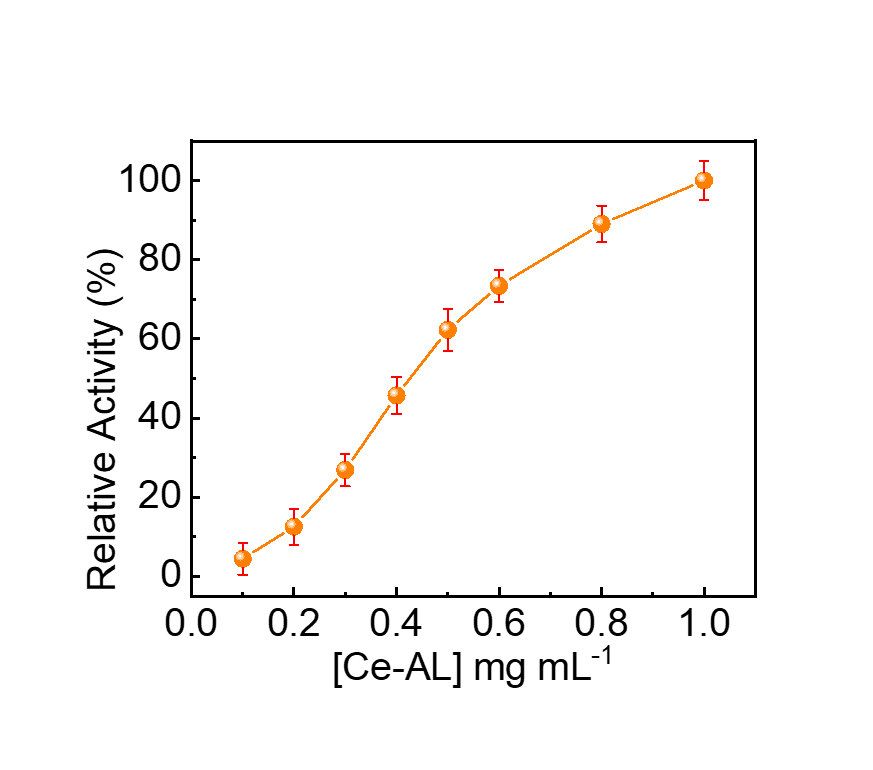


Figure S5. The effects of Ce-AL dosages on the colorimetric systems. Error bars represent the standard deviation from five independent experiments.


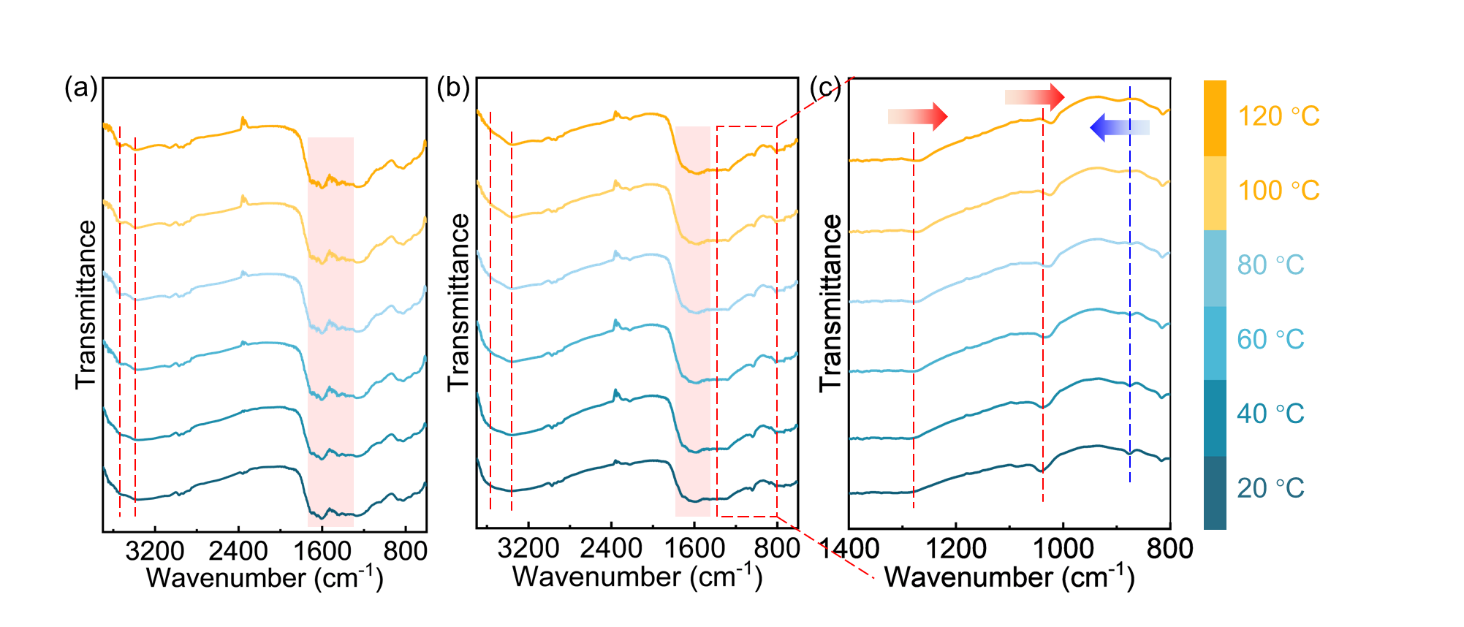


Figure S6. DRIFT spectra of (a) aminated lignin and (b) Ce-AL recorded at temperatures ranging from 20 to 120 ºC. (c) An enlarged view of (b) in the range of 1400 to 800 cm^-1^.


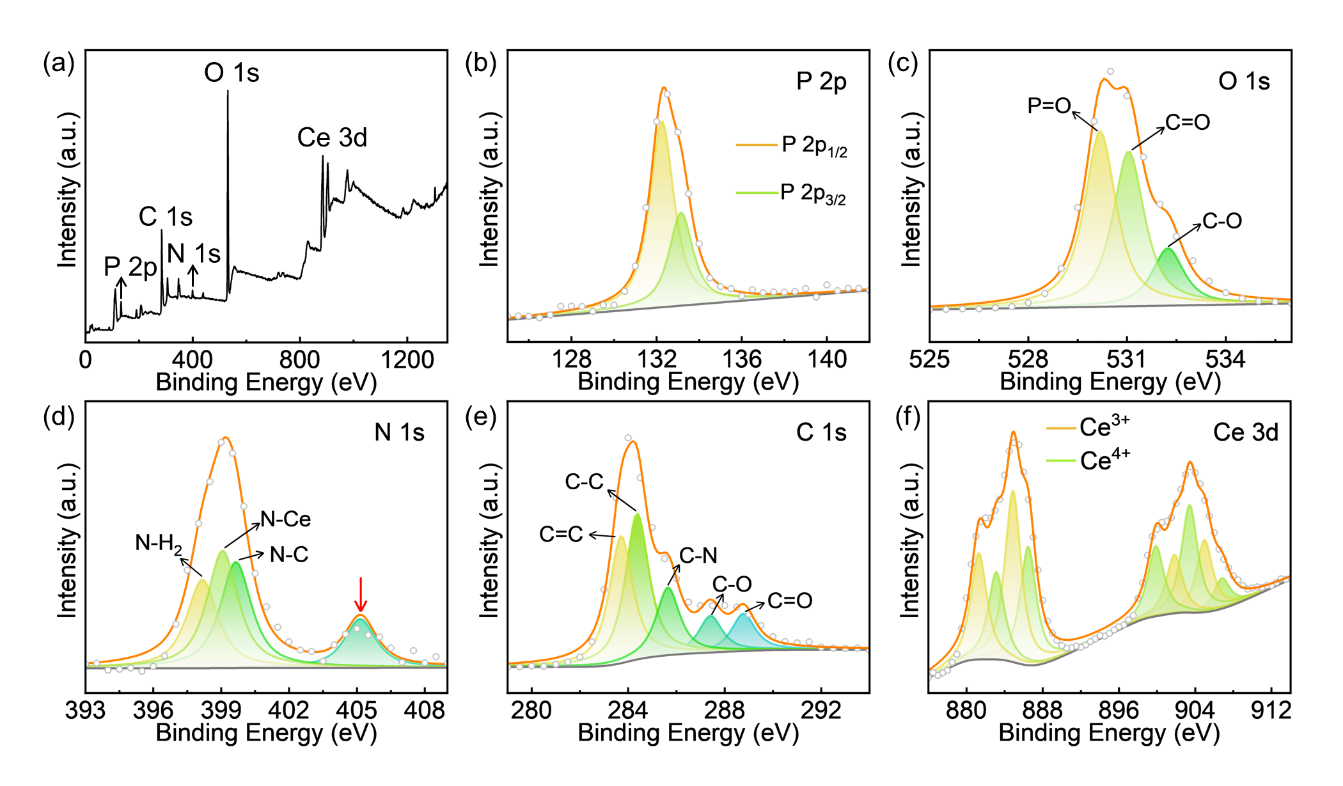


Figure S7. (a) XPS survey scan and high-resolution spectra of (b) P 2p, (c) O 1s, (d) N 1s, (e) C 1s and (f) Ce 3d of Ce-AL after 1-time use (Ce-AL-U1).


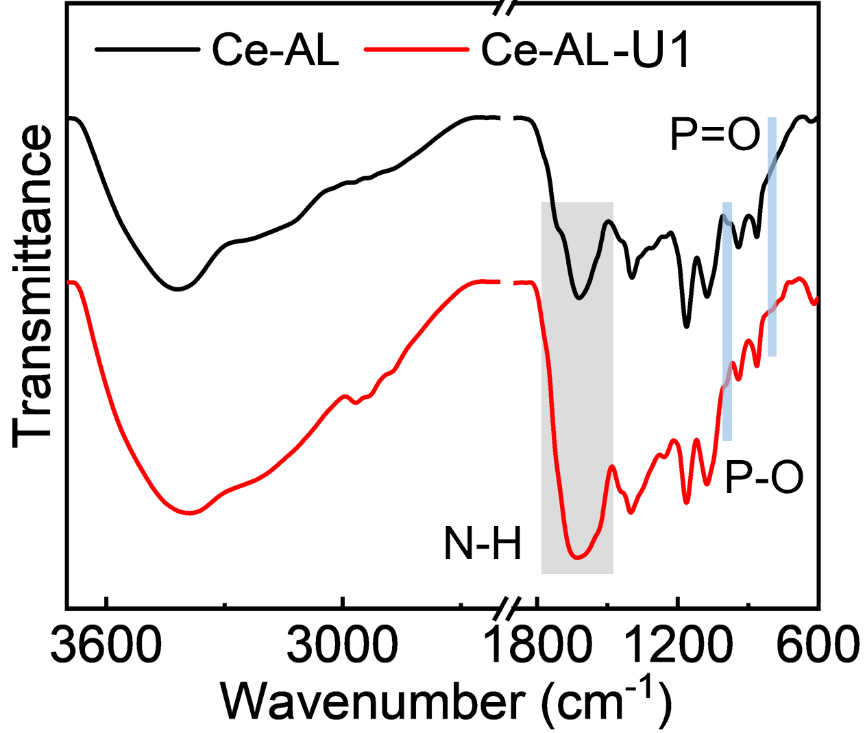


Figure S8. FT-IR spectra of Ce AL and Ce-AL-U1.


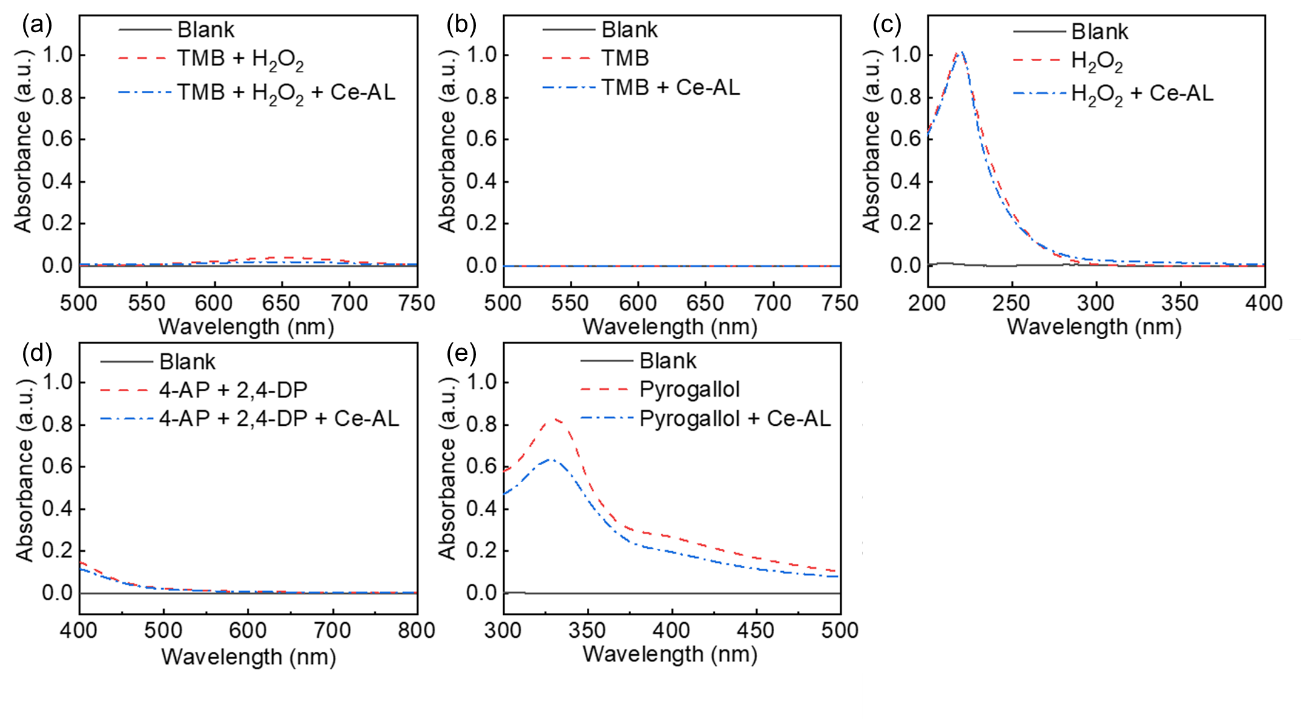


Figure S9. (a) Peroxidase-, (b) oxidase-, (c) catalase-, (d) laccase-, and (e) superoxide dismutase-like activities of Ce-AL sample.

**
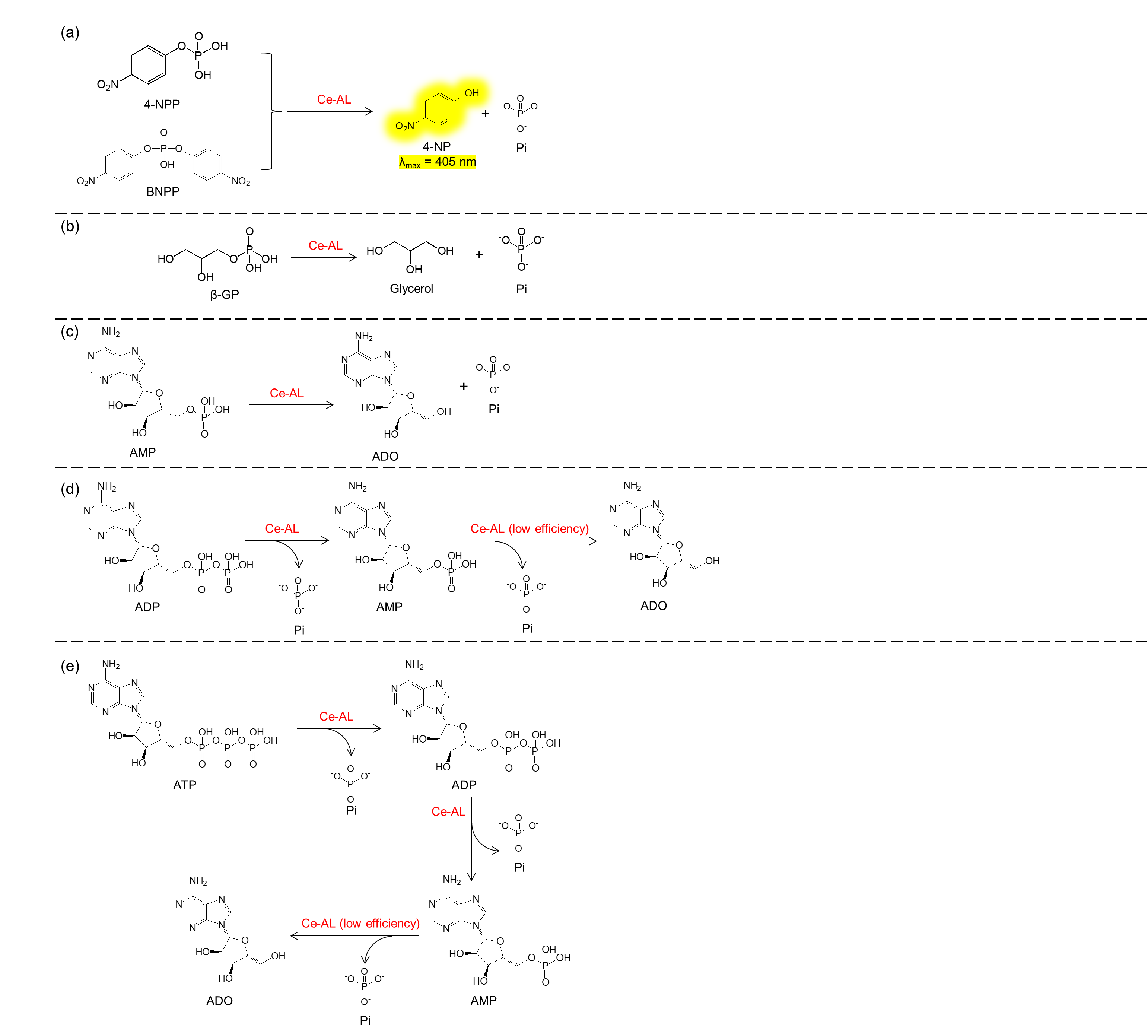
**

Figure S10. The chemical reaction equations of (a) 4-NPP and BNPP, (b) β-GP, (c) AMP, (d) ADP, and (e) ATP hydrolyzed by Ce-AL.


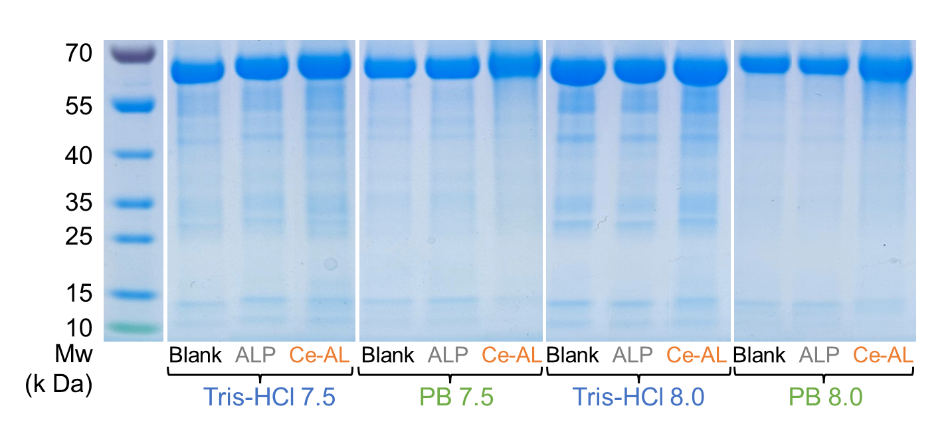


Figure S11. The SDS-PAGE patterns of BSA before and after hydrolysis using Ce-AL and ALP in phosphate and Tris-HCl buffer systems at different pH values.

**
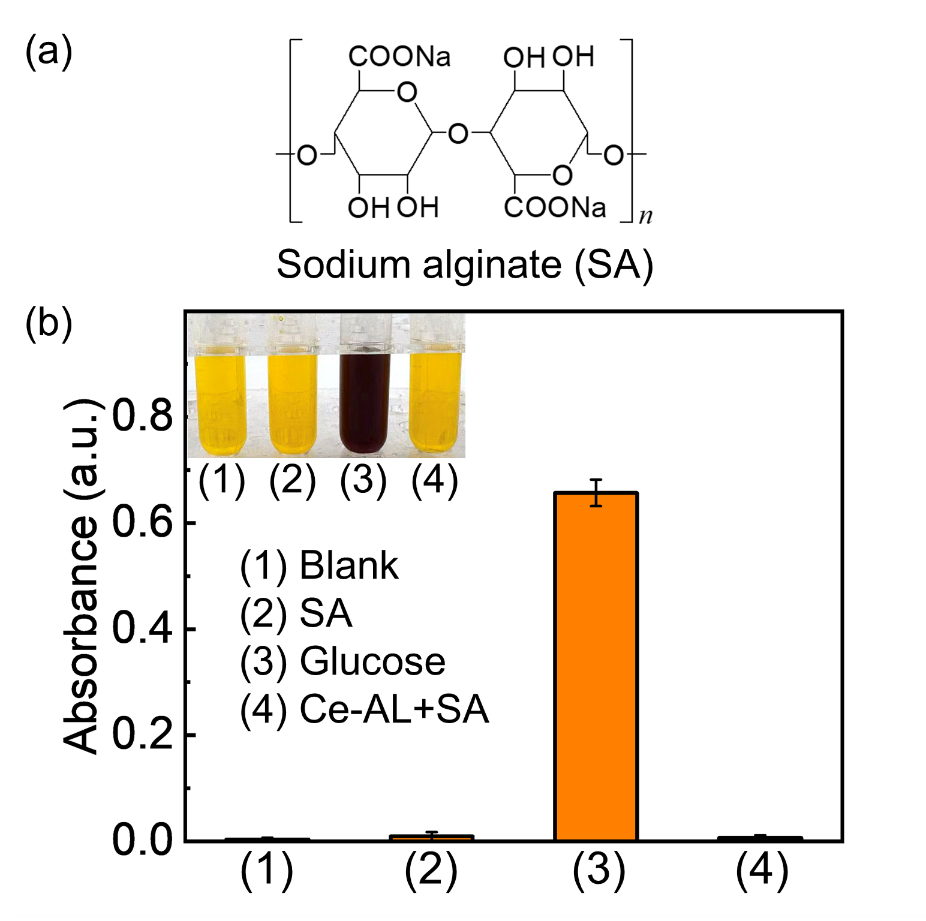
**

Figure S12. (a) Chemical structures of sodium alginate; (b) Absorbance at 540 nm of (1) blank control, (2) SA, (3) glucose, and (4) Ce-AL treated SA after reacted with DNS reagent and diluted for 10 times. Error bars represent the standard deviation from five independent experiments.


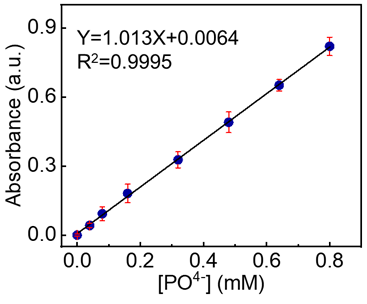


Figure S13. Standard curve line for quantifying inorganic phosphate (Pi) using molybdenum blue method. Error bars represent the standard deviation from five independent experiments.

**
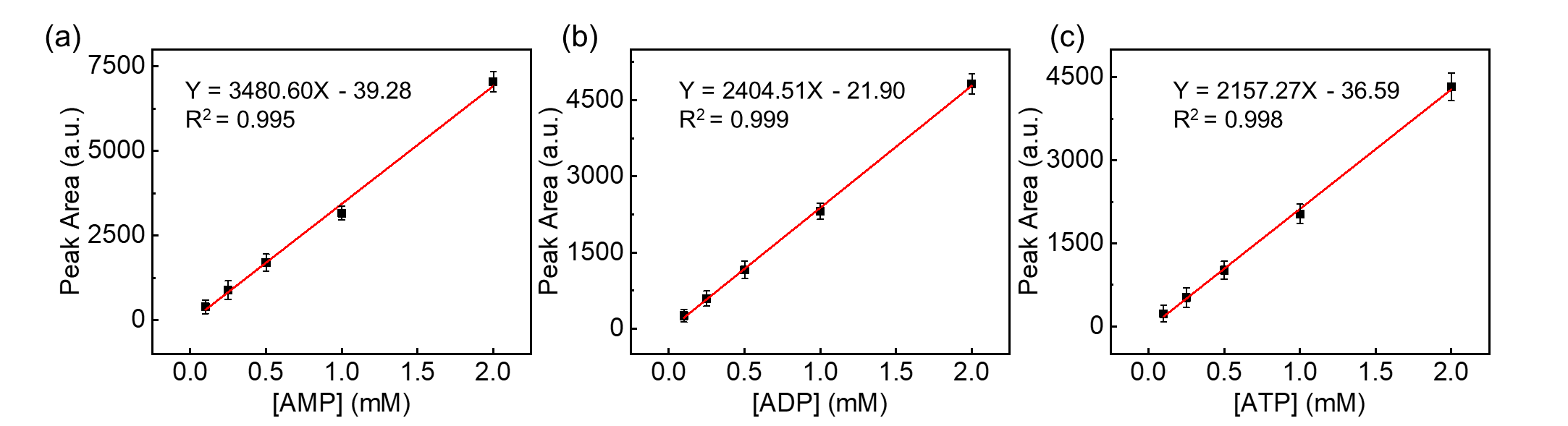
**

Figure S14. The standard curves of peak areas as a function of (a) AMP, (b) ADP, and (c) ATP standard substrates concentrations, as measured by liquid chromatography. Error bars represent the standard deviation from five independent experiments.

**3. Supporting tables：**

Table S1. Element content of cerium in Ce-AL synthesized with different qualities of cerium sources.

| Samples | Cerium contents (wt%) |
| --- | --- |
| Ce-AL-0.25 | 6.24 |
| Ce-AL-0.5 | 6.33 |
| Ce-AL-1.0 | 9.73 |
| Ce-AL-1.5 | 10.04 |

Table S2. The catalytic parameters of different catalysts on 4-NPP.

| Catalysts | K_m_ (mm) | V_max_ (m s^-1^) | K_cat_ (s^-1^) | Ref. |
| --- | --- | --- | --- | --- |
| DCNG | 0.029 | 7.170×10^-7^ | 73.213 | ^[^[^2^](#_ENREF_2)^]^ |
| Ce-MOF | 0.252 | 9.860×10^-7^ | 2.310 | ^[^[^3^](#_ENREF_3)^]^ |
| MOF_-2.5Au-Ce_ | 0.876 | 2.060×10^-7^ | 343.333 | ^[^[^4^](#_ENREF_4)^]^ |
| ZIF-90 | 1.950 | 4.000×10^-9^ | 7×10^-4^ | ^[^[^5^](#_ENREF_5)^]^ |
| Hf-Ni | 0.230 | 2.783×10^-8^ | 1×10^-4^ | ^[^[^6^](#_ENREF_6)^]^ |
| P2-Zn(II) | 0.930 | 4.000×10^-8^ | 7×10^-4^ | ^[^[^7^](#_ENREF_7)^]^ |
| SA Ce-N-C | 0.276 | 5.855×10^-6^ | - | ^[^[^8^](#_ENREF_8)^]^ |
| Natural ALP | 0.160 | 3.917×10^-7^ | - | This work |
| Ce-AL | 0.200 | 1.085×10^-8^ | 0.001 | This work |

Table S3. Optimal pH of different substrates catalyzed by Ce-AL and natural ALP.

| Substrates | Optimal pH of  Ce-AL-containing systems | Optimal pH of natural  ALP-containing systems |
| --- | --- | --- |
| 4-NPA | 7.0 | 7.0 |
| BNPP | 10.0 | 10.0 |
| AMP | 9.0 | 8.0 |
| ADP | 10.0 | 10.0 |
| ATP | 10.0 | 10.0 |
| β-GP | 10.0 | 7.5 |
| 4-NPP | 9.0 | 10.0 |

Table S4. Experimental conditions for evaluating the peroxidase-, oxidase-, catalase-, superoxide dismutase-, and hydrolase-like activities of Ce-AL.

| Enzyme-like activities | Blank groups | Control groups | Experimental groups | Reaction time (min) |
| --- | --- | --- | --- | --- |
| Peroxidase | NaAc-HAc,  pH 5.0 | TMB (0.2 mM);  H_2_O_2_ (100 mM) | Ce-AL (1 mg mL^-1^);  TMB (0.2 mM);  H_2_O_2_ (100 mM) | 30 |
| Oxidase | NaAc-HAc,  pH 5.0 | TMB (0.2 mM) | Ce-AL (1 mg mL^-1^);  TMB (0.2 mM) | 30 |
| Catalase | Tris-HCl,  pH 7.0 | H_2_O_2_ (10 mM) | Ce-AL (1 mg mL^-1^);  H_2_O_2_ (10 mM) | 30 |
| Laccase | Tris-HCl,  pH 7.0 | 4-aminoantipyrine  (2 mM);  2,4-dichlorophenol  (2 mM) | Ce-AL (1 mg mL^-1^);  4-aminoantipyrine (2 mM);  2,4-dichlorophenol (2 mM) | 30 |
| Superoxide dismutase | Tris-HCl,  pH 8.0 | Pyrogallol  (35 μg mL^-1^) | Ce-AL (1 mg mL^-1^);  pyrogallol (35 μg mL^-1^) | 5 |
| Hydrolase | diethanolamine-HCl,  pH 9.0 | 4-nitrophenyl phosphate  (2 mg mL^-1^) | Ce-AL (1 mg mL^-1^);  4-nitrophenyl phosphate (2 mg mL^-1^) | 30 |

**Reference**

[1] B. Adney, J. Baker, Measurement of Cellulase Activities, **1996**.

[2] Y. Xiong, L. Su, Y. Peng, S. Zhao, F. Ye, Dextran-coated Gd-based ultrasmall nanoparticles as phosphatase-like nanozyme to increase ethanol yield via reduction of yeast intracellular ATP level, *J. Colloid Interface Sci.* **2022**, *627*, 405-414, https://doi.org/10.1016/j.jcis.2022.07.036.

[3] X. Yuan, J. Xiong, X. Wu, N. Ta, S. Liu, Z. Li, W.-Y. Lou, Ultrasmall Ce-based metal–organic frameworks nanozyme with hydrolytic activity for boosting antibiofilm therapy, *Chem. Eng. J.* **2024**, *480*, 148246, https://doi.org/10.1016/j.cej.2023.148246.

[4] Z. Liu, F. Wang, J. Ren, X. Qu, A series of MOF/Ce-based nanozymes with dual enzyme-like activity disrupting biofilms and hindering recolonization of bacteria, *Biomaterials* **2019**, *208*, 21-31, https://doi.org/10.1016/j.biomaterials.2019.04.007.

[5] T. Fu, C. Xu, R. Guo, C. Lin, Y. Huang, Y. Tang, H. Wang, Q. Zhou, Y. Lin, Zeolitic Imidazolate Framework-90 Nanoparticles as Nanozymes to Mimic Organophosphorus Hydrolase, *ACS Appl. Nano Mater.* **2021**, *4* (4), 3345-3350, https://doi.org/10.1021/acsanm.1c00540.

[6] J. Dong, H. D. An, Z. K. Yue, S. L. Hou, Y. Chen, Z. J. Zhang, P. Cheng, Q. Peng, B. Zhao, Dual-Selective Catalysis in Dephosphorylation Tuned by Hf(6)-Containing Metal-Organic Frameworks Mimicking Phosphatase, *ACS Cent. Sci.* **2021**, *7* (5), 831-840, https://doi.org/10.1021/acscentsci.0c01581.

[7] J. Yang, Z. Wang, W. Xiao, Y. Peng, M. Qiu, X. Xiong, Y. Lu, T. Chen, Z. Xu, Zn(II)-complex-based hydrolytic nanozymes prepared by nanoprecipitation for the hydrolysis of carboxylic esters and methyl parathion, *Colloids Surf., A* **2023**, *675*, 132034, https://doi.org/10.1016/j.colsurfa.2023.132034.

[8] G. Song, J. C. Li, Z. Majid, W. Xu, X. He, Z. Yao, Y. Luo, K. Huang, N. Cheng, Phosphatase-like activity of single-atom CeNC nanozyme for rapid detection of Al(3), *Food Chem.* **2022**, *390*, 133127, https://doi.org/10.1016/j.foodchem.2022.133127.
